# Supplementary material for: An incentive circuit for memory dynamics in the mushroom body of Drosophila melanogaster
Source: eLife. 2022 Apr 1;11:e75611. doi: 10.7554/eLife.75611 (PMC8975552; doi:10.7554/eLife.75611)
Supplement: Transparent reporting form [file elife-75611-transrepform1.pdf]

Westbrook Centre
Milton Road
Cambridge CB4 1YG, UK
P 01223 855340
W elifesciences.org
T @elife

eLife's transparent reporting form

We encourage authors to provide detailed information *within their submission* to facilitate the interpretation and replication of experiments. Authors can upload supporting documentation to indicate the use of appropriate reporting guidelines for health-related research (see EQUATOR Network), life science research (see the BioSharing Information Resource), or the ARRIVE guidelines for reporting work involving animal research. Where applicable, authors should refer to any relevant reporting standards documents in this form.

If you have any questions, please consult our Journal Policies and/or contact us: editorial@elifesciences.org.

Sample-size estimation

- You should state whether an appropriate sample size was computed when the study was being designed
- You should state the statistical method of sample size computation and any required assumptions
- If no explicit power analysis was used, you should describe how you decided what sample (replicate) size (number) to use

Please outline where this information can be found within the submission (e.g., sections or figure legends), or explain why this information doesn't apply to your submission:

> No statistical methods were used to predetermine sample size. For Figures 4-8 and their figure supplements, we chose sample sizes that were comparable to similar experiments conducted by others on the field. Regarding Figure 11 and its figure supplements, we empirically choose 100 samples for each condition and every experiment to achieve better confidence interval. The number of samples in all cases are indicated in the figure captions.

Replicates

- You should report how often each experiment was performed
- You should include a definition of biological versus technical replication
- The data obtained should be provided and sufficient information should be provided to indicate the number of independent biological and/or technical replicates
- If you encountered any outliers, you should describe how these were handled
- Criteria for exclusion/inclusion of data should be clearly stated
- High-throughput sequence data should be uploaded before submission, with a private link for reviewers provided (these are available from both GEO and ArrayExpress)

Please outline where this information can be found within the submission (e.g., sections or figure legends), or explain why this information doesn't apply to your submission:

> The data collection process for Figures 4-8 and their figure supplements is described in section "Data collection". The processing of these data is described in section "Calculating off- and on-shock values", and the script used for this processing and visualization is in part of the code repository in https://github.com/InsectRobotics/IncentiveCircuit.

eLife Sciences Publications, Ltd is a limited liability non-profit non-stock corporation incorporated in the State of Delaware, USA, with company number 5030732, and is registered in the UK with company number FC030576 and branch number BR015634 at the address Westbrook Centre, Milton Road, Cambridge CB4 1YG.

Statistical reporting

- Statistical analysis methods should be described and justified
- Raw data should be presented in figures whenever informative to do so (typically when N per group is less than 10)
- For each experiment, you should identify the statistical tests used, exact values of N, definitions of center, methods of multiple test correction, and dispersion and precision measures (e.g., mean, median, SD, SEM, confidence intervals; and, for the major substantive results, a measure of effect size (e.g., Pearson's r, Cohen's d)
- Report exact p-values wherever possible alongside the summary statistics and 95% confidence intervals. These should be reported for all key questions and not only when the p-value is less than 0.05.

Please outline where this information can be found within the submission (e.g., sections or figure legends), or explain why this information doesn't apply to your submission:

We use Pearson's correlation coefficient and the p-value in Figure 3-Figure supplement 1 and "The incentive circuit" section in order to directly compare our result to the original work of the experiment (Bennett et al., 2021). For the rest of our results, we have not used statistical significance analysis. Statistical significance could be used only for the behavioural results presented in Figure 11 (and figure supplements). However, the fact that this is a behaviour produced by a computational model allows for conclusions to emerge from more sophisticated methods.

(For large datasets, or papers with a very large number of statistical tests, you may upload a single table file with tests, Ns, etc., with reference to sections in the manuscript.)

Group allocation

- Indicate how samples were allocated into experimental groups (in the case of clinical studies, please specify allocation to treatment method); if randomization was used, please also state if restricted randomization was applied
- Indicate if masking was used during group allocation, data collection and/or data analysis

Please outline where this information can be found within the submission (e.g., sections or figure legends), or explain why this information doesn't apply to your submission:

Not applicable.

Additional data files ("source data")

- We encourage you to upload relevant additional data files, such as numerical data that are represented as a graph in a figure, or as a summary table
- Where provided, these should be in the most useful format, and they can be uploaded as "Source data" files linked to a main figure or table
- Include model definition files including the full list of parameters used
- Include code used for data analysis (e.g., R, MatLab)
- Avoid stating that data files are "available upon request"

Please indicate the figures or tables for which source data files have been provided:

All data generated or analysed during this study are included in the manuscript and supporting files. Data used for Figure 3-Figure supplement 1 can be found in Figure 3-source data 1, and data used for Figures 4-8 can be found in Figure 4-source data 1. The scripts for producing the data and generating Figures 6 (C, D & E), 7 (D & E), 8 (C & D), 11B, 12, 16 (A & B) and all figure supplements are located at https://github.com/InsectRobotics/IncentiveCircuit. Figures 5A, 6A, 7A, 7C, 8A, 9A, 9B and Figure 5-Figure supplement 1A were generated using the Fly Brain software. All figures were edited using the Inkscape software.

eLife Sciences Publications, Ltd is a limited liability non-profit non-stock corporation incorporated in the State of Delaware, USA, with company number 5030732, and is registered in the UK with company number FC030576 and branch number BR015634 at the address Westbrook Centre, Milton Road, Cambridge CB4 1YG.
