# Supplementary material for: An incentive circuit for memory dynamics in the mushroom body of Drosophila melanogaster
Source: eLife. 2022 Apr 1;11:e75611. doi: 10.7554/eLife.75611 (PMC8975552; doi:10.7554/eLife.75611)
Supplement: Peer review history

# Editor report: Editor's evaluation

**DOI:** 10.7554/eLife.75611.sa0

**Authors:** Upinder Singh Bhalla

**Affiliations:** https://ror.org/03ht1xw27 Tata Institute of Fundamental Research India

This ambitious study goes from signalling mechanisms to fly behavior through a model of a memory circuit in the fly brain. The authors call this the incentive circuit. The model draws extensively from anatomical and physiological measurements. The study makes a wide range of predictions about how this circuit mediates behaviour and learning through attractive and repulsive cues.

# Decision letter

**DOI:** 10.7554/eLife.75611.sa1

**Affiliations:** https://ror.org/03ht1xw27 Tata Institute of Fundamental Research India; https://ror.org/02tyrky19 Trinity College Dublin Ireland

In the interests of transparency, eLife publishes the most substantive revision requests and the accompanying author responses.

**Decision letter after peer review:**

[Editors’ note: the authors resubmitted a revised version of the paper for consideration. What follows is the authors’ response to the first round of review.]

Thank you for submitting the paper "The incentive circuit: memory dynamics in the mushroom body of *Drosophila melanogaster*" for consideration by *eLife*. Your article has been reviewed by 3 peer reviewers, one of whom is a member of our Board of Reviewing Editors, and the evaluation has been overseen by a Senior Editor. The following individual involved in review of your submission has agreed to reveal their identity: Mani Ramaswami (Reviewer #3).

We are sorry to say that, after consultation with the reviewers, we have decided that this work will not be considered further for publication by *eLife*.

While all the reviewers appreciate the ambition and value in integrating diverse sources of data to developing a model of learning, they had some substantial concerns. These are elaborated in their detailed comments, and I provide a distillation of the discussion that the reviewers and I had about the paper. Since it will take considerable further work to address these points, the reviewers and I felt that the paper should be rejected. If the authors wish to resubmit after completely addressing the concerns this would be fine.

1. The reviewers found the paper a difficult read. Could the authors rewrite to make it accessible to a wide range of readership?

2. The formulation of the DLR seems to be a variant of RPE (Reward Prediction Error) learning rules, and hence the conclusions need to be re-evaluated.

Can the authors re-think the basic formulation of DLR starting with Equations (2) and (3)? There should be some experimental tests if the DLR is indeed determined to be different from regular RPE.

3. The microcircuits should be better based on experimental data. From our understanding, the data shown in Figures 4H/G, 5B/C/E/F and 6B/C seems to have been obtained by simulations. Would Ca recordings for these figures be feasible? Can there be stronger justification for the connectivity of the proposed incentive circuit?

3b. The proposed circuit connectivity of the 'incentive circuit' needs to be defined for each MBON because most contemporary work shows that different kinds of memory involved plasticity in different subsets of MBONs. Can the model make specific testable predictions for each subset of MBON?

4. Further experimental predictions should be made, based on well-parameterized models of the underlying neurons. Can the authors provide considerably more clarity on which sets of behavioral or physiological data are selected by the authors as targets or tests for specific parts of their model?

5. Can the model account for existing data showing overlapping conflicting engrams? Additional experiments and simulations may be needed to ascertain this.

*Reviewer #1 (Recommendations for the authors):*

This ambitious study builds a model of a proposed key circuit motif in fly behaviour and learning, the Incentive Circuit. The authors examine its implications for a variety of behaviours, and perform a thorough circuit-level mapping of model neuronal activity to recordings. The model uses abstracted model neurons and synaptic signaling, but with careful attention to experimental data at many steps. The mapping to experiments is good, and the model makes far-reaching predictions for animal behaviour.

The development of the model is generally well presented. The learning rule is derived from earlier work (Handler et al) and then the authors transform the terms for ER-ca^2+^ and for cAMP to terms emerging from DA inputs. The model development is especially systematic, building up to the final version step by step with reference to experiments. Importantly, these are mapped to specific sets of experimental observations on the circut level.

I have mostly comments to clarify or strengthen the presentation.

1. I had a little trouble to envision the two components of D2 and D1. Are they time-varying? Seems to be, see Equation 4, where they are presented as D1(t) and D2(t). In other words, do they express D2 and D1 as distinct α functions following spike activity in the DAN?

However, in the text and figures it is frequently presented in terms such as D2 > D1 (eg., Figure 3), which looks like a static effect. This was confusing.

Also in Figure 2A, are we seeing the peak values of ER-Ca or area under curve?

Around line 128 it is a hint that it is area under curve, but I am not sure.

2. I would have liked to have seen some more mapping to functional experiments in the figures up to Figure 7, where the components of the model are being built up. The authors mention several in the text. Even a qualitative look at the experimental responses would help to strengthen the motivation of the model design.

3. The authors then utilize this circuit in an aversive olfactory conditioning paradigm, for which they provide experimental data corresponding to the various neuron types. They then simulate this. This is an outstanding way to validate/test their model. It would be helpful to have the experimental and simulated responses interleaved on the same figure so as to better compare.

4. I appreciate that it is quite challenging for a simulation to simultaneously replicate properties of several intermediate stages of circuit activity, even more so when the stimulus is not one that the model has been trained on. Could the authors confirm that this is indeed the case, i.e. that the model outcome for figure 9 was obtained only from the parameter tuning earlier in the paper up to Figure 7?

5. It would be useful to perform a statistical evaluation of the fidelity of the model as compared to experiment.

6. The authors then place their model flies in a virtual arena and explore a number of behaviours. Here they contrast their model behaviour with the predictions from a different learning, reward prediction error. I would have liked to have seen in figure 11 an illustration of the correspondence to experimental observations from the literature.

*Reviewer #2 (Recommendations for the authors):*

The manuscript in its current form is built around two main threads. In the first thread, the authors review several results in the literature on associative learning in the mushroom body of the adult fruit fly, and construct an Incentive Circuit (IC) consisting of 6 dopaminergic and 6 mushroom body neurons with specific memory dynamics. They provide a coherent functional view of some of the disparate recent results in associative learning of the mushroom body.

The second thread incorporates a Dopaminergic Learning Rule (DLR) into the IC computational model, providing a computational system for evaluating the learning mechanisms involved.

A weakness here is that the acquisition, forgetting and assimilation of memories qualitatively described in the first thread are not strongly linked with the quantitative IC model described in the second thread.

Conversely, the validation of the IC model circuit, given the noisy data that the authors provide, is only possible in terms of trends, i.e., simple visual inspection. Interpreting the data then is difficult as it does not provide enough constraints for the computational model.

Given the limitations inherent in the validation of the IC from their recorded data, the authors proceed to explore the DLR using behavioral experiments purely based on simulations. This is an effective methodology widely employed in, e.g., robotics. The authors extensively compare the 'learning/navigation' performance of DLR with a variant of reward prediction error (RPE) learning rule and demonstrate a better learning performance. While the comparison may be compelling, we found that underlying the DLR, is the computation of a prediction error, i.e., DLR is a variant of RPE. This calls for a re-evaluation, positioning and clarification of some of the key conclusions regarding why the DLR is effective in associative learning tasks.

l.128 The section 'Mushroom Body Microcircuits' makes good first reading. However, most of the key statements could further benefit from more extensive quantitative backing as hinted at in Figures 4, 5 and 6 (see also my comment below). Since these microcircuits are simpler than the IC, my expectation is that they could provide better intuition regarding their function.

Figures 4F and 4G are rather difficult to understand/parse. More caption details, choice of different colors, would help.

Same comment regarding Figures 5B, 5C, 5E and 5F, and 6B, 6C.

While Figure 8 is to be commended, the data is rather noisy and, in my view, despite the best intentions, rather difficult to understand/evaluate. As the authors argue in l.312, 'we computationally modelled the incentive circuit in order to demonstrate all the properties we described before and compare the reconstructed activities to the ones of Figure 8C'. However, a comparison by simple visual inspection is rather unconvincing. The need for introducing a distance measure is in order.

I found 'modeling behavior', as presented in the current version of the manuscript, to be quite effective. However, I'd like to note that in the process, the authors changed the underlying PN activity model. This requires, given that the rest of the paper is based on a binary odor model of the PN activity (see the discussion preceding Equation (6)), some careful/detailed assessment of its implications. Finally, the authors propose to compare their DLR with a variant of RPE. Here a major conceptual problem arises.

The authors argue that DLR is a fundamentally different learning rule from RPE. They state in l.462 that 'The idea behind RPE is that the fly learns to predict how rewarding or punishing a stimulus is by altering its prediction when this does not match the actual reward or punishment experience'.

This can be adapted to the mushroom body circuit by assuming that the MBON output provides a prediction of DAN activity. But this is exactly what Equation (18) states. The differential equation (18) describing the gradient of the DAN activity is equal to sum of the weighted shock delivery ('transform' in l.750) and the weighted MBON activity (l.755).

The sum is just the prediction error between the two terms. Consequently, since the DLR is, in view of this reviewer, a variant of RPE, a comparison with another RPE is of little interest. A substantial re-write of the paper starting with the section on the Incentive Circuit (l. 257) is in order.

l.765: "The above matrices summarise the excitatory (positive) and inhibitory (negative) connections between MBONs and DANs or other MBONs. The magnitude of the weights was hand-tuned in order to get the desired result." This 'hand-tuning" appears, to me, to be a 'construction' of the prediction error on the right hand side of Equation (18). Some details might help clarify to what extent the hand-tuning is based on the assumptions of the binary model of the 2 odors at the PN level. I presume that the generality of the model alluded to in l.743 stating that 'that the number of neurons we are using for PNs and KCs is not very important and we could use any combination of PN and KC populations' breaks down and the hand-tuning needs to be repeated every time the number of neurons is changed.

*Reviewer #3 (Recommendations for the authors):*

The authors propose an original dopaminergic learning rule, which, when implemented in simple neural circuit motifs shown to exist within the *Drosophila* mushroom body (MB) , can potentially account for a very large number of independent, poorly integrated physiological and behavioural phenomena associated with the mushroom body. It considers multiple behavioural roles of MB output neurons beyond attraction and aversion and offers new insight to the how the MB functions in acquisition, consolidation and forgetting of memories. The manuscript further attempts to show how similar principles could potentially be useful in the mammalian brain. An ambitious and integrative analysis of this sort is sorely needed in the field.

The paper has obviously involved very broad and deep consideration of the MB connectome as well as genetic, physiological and behavioural studies of the roles of the different classes of Kenyon cells, MBONs and DANs that innervate the mushroom body. It is original and ambitious and potentially very valuable to the field.

My major reservation is that the manuscript is very difficult to read and evaluate by anyone who is not a *Drosophila* mushroom body aficionado. I consider myself an interested reader and one who keeps broad track of the field, but found the need to read and evaluate far too many papers cited by the authors to decide how well phenomena the authors attempt to model have been demonstrated and how well assumptions made by the authors are justified by data. E.g. I was stymied even at figure 1, where mutual inhibition between MBONs is indicated and it took me considerable (and eventually futile) effort to look into where and how well this has been established.

To make the work more accessible at least to this moderately educated reviewer, I fear that a major re-rewrite will be required. I would suggest that for each section – exactly has been shown be clearly enumerated, with enough detail provided for the reader to judge the strength of these data. The justification and support for three types of MBONs and their incentive should also be particularly clearly indicated.

Moreover, while the authors are correct to point out the limitations of current models based on dopamine prediction-error, I do wonder if there is room for prediction error to also contribute meaningfully within the framework proposed in this paper.

I apologise for the not having a list of specific issues for the authors to address, because I found the basis to be so difficult to explore but here is some general feedback.

1. It is nice that and the dynamics of neural responses obtained with the model correspond closely with ones reported in previous studies (although there are exceptions, some nicely highlighted by the authors).

2. There should be deeper engagement with signalling mechanisms that differentiate the two types of dopamine receptors. I found the assumptions regarding their differences to be useful for the modelling of different effects of reinforcement before or after sensory experience (Ruta Cell 2019), but quite superficial in terms of providing hypothesis for how the receptors may differ in terms of mechanism of action.

3. ON the same note, specific experimental predictions of the model could also be clearly indicated at the end of each section.

4. While the authors admittedly designed informative and clear figures, and their Table 1 points the reader to papers that report relevant neural connections and neuronal functions, this is not enough. Data in support of each assumption should be clearly and specifically mentioned and hypotheses connections also clearly stated. After considerable effort, I still could find no evidence for lthe existence of inhibitory connections between MBONγ4 and MBONγ2 (which is not to say that none exist – but surely it is the authors job to clarify this).

5. The authors should also try to account for the discovery of parallel, independent memory traces (like appetitive LTM formation towards the CS- in classic LTM aversive training paradigms).

6. Does the dopaminergic learning rule explain the differences in dynamics and memory strength between appetitive and aversive memories? These two types of memory involved different molecular components and display different learning rules (stronger short-term aversive memories and longer-lasting appetitive memories requiring less training)? This should perhaps be clarified, particularly since KC output appears dispensable for aversive learning (aquisition) but potentially necessary for the acquisition of appetitive memories (Pribbenow et al., 2021).

7. I found the easy assumption that forgetting involves erasure to be troubling. Perhaps this happens sometimes. But many apparently "forgotten" memories are never erased, simply not reactivated for multiple reasons. Intellectually this point needs to be acknowledged.

[Editors’ note: what follows is the authors’ response to the second round of review.]

Thank you for resubmitting your work entitled "The incentive circuit: memory dynamics in the mushroom body of *Drosophila melanogaster*" for further consideration by *eLife*. Your revised article has been evaluated by Ronald Calabrese (Senior Editor) and a Reviewing Editor.

The manuscript has been improved but there are some remaining issues that need to be addressed, as outlined below:

1. Could the authors compare their simulated/predicted behavior with some quantitative or semi-quantitative measures of experimental behavior?

2. Can the authors elaborate on their mapping of ER-CA and cAMP in the model with the cited data? This relates to point 4 from Reviewer 1.

3. Can the authors do some parameter sensitivity analysis as suggested by the reviewers?

In addition, the reviewers had a few points for the authors to expand upon in the revision, and a number of useful suggestions to improve clarity.

*Reviewer #1 (Recommendations for the authors):*

This is an ambitious but also highly complicated odelling study that seeks to account for a wide range of fly learning behaviour in terms of underlying learning rules and circuitry.

The strengths of the study are its ambition, detail and substantial attention to experimental inputs. In principle it builds up a large and testable conceptual framework for understanding many aspects of learning. Its weaknesses, which are readily fixed, are 1. That the study misses opportunities to better compare model to experiments. And 2, that the study doesn’t do a systematic parameter and model exploration to see how robust are the properties.

With these additions the study would be strong and of value to the field in laying out a template for further investigation. The authors posit that this framework could also apply to other organisms.

1. This is an ambitious but also highly complicated odelling study that seeks to account for a wide range of fly learning behaviour in terms of underlying learning rules and circuitry. The authors have made substantial improvements to the clarity of the presentation, particularly with regards to comparison of experimental and simulated data.

I would have liked to see similar comparison for two more features: the behaviour, and the crucial learning rule section, as I comment below. I note that a similar request was made in an earlier review.

2. The other big thing I would have liked to see is an exploration of parameter sensitivity. This is needed both because of model complexity and because of the not-perfect match between model and input data. No model is perfect, but the confidence in a model is much improved if one can see that it still ‘works’ even when the numbers (and other assumptions) shift around a bit.

3. Behaviour: The authors have made the interesting and potentially powerful step of linking their model to measurable behaviour. But they miss the opportunity to put the outcomes (experiment and model) side by side. Even a semi-quantitative distillation to some common metric for displaying and comparing the experimental and model properties would have been valuable.

4. Figure 16: Details of ER-CA and cAMP in the model don’t match data. The form of the pairing for ER Ca is inconsistent with the data of Handler et al., particularly when CS precedes US by a large interval. Handler et al. show no response for forward pairing even several seconds after the last stimulus. Also, the time-course of ER response for the backward pairing case is inconsistent. In the Handler data (Figure 6) the ER signal remains low (i.e, very different from baseline) well past 5 seconds, whereas in Figure 16 the signal returns to baseline within 5s. I am also concerned that there doesn't seem to be experimental support for the reduced cAMP signal at very small overlap intervals. Indeed, the Handler data suggests that there is a large signal at the 0.5s and -1.2s points. Figure 16 shows that the model assumes a low and brief signal at -1.2s.

I would have appreciated having the experimental data from Handler and others illustrated here in the same figure, just to see how well the model forms behave. It would save the reader the step of going to look up another paper and tracking down appropriate figure panels.

5. As one example of a useful parameter sensitivity analysis: The form of the deltaWij seems rather crucial to the model, so I'm homing in on this. It is a difference of two values which are themselves clearly the difference of opposing signals. It would therefore be valuable to show that relaxation of these tight timing requirements does not upset the learning rule and subsequent behaviour.

It would be useful to see similar sensitivity analyses for other key parts of the model.

Clarifications:

6. pg 28: 3 lines from bottom.

Do the authors mean "activity of the ith presynaptic KC? 'Target' sounds like it is postsynaptic.

7. Equation 30 onward.

w_rest: Is this a global parameter for all synapses?

w_rest: The way it is used in the equation looks more like a_rest, the resting activity of the synapse. Sorry to be pedantic, but the units of weight and rate don't match.

This gets further mixed in the equation between lines 853 and 854 where the authors add ki and Wij. Maybe ki is scaled somehow to weights?

8. Figure 5 and later: The responses, both experimental and model, are shown as an up-down oscillation. I assume that the up states are measurements during the training, and down is measurement half a day later. But this is hard to see from the text or legends, and I had to go down to the last section in the methods to see that this seems to be described as on-shock and off-shock values. It is confusing and should be mentioned in the figure legends and accompanying text.

*Reviewer #2 (Recommendations for the authors):*

The authors propose an original dopaminergic learning rule, which, when implemented in simple neural circuit motifs shown to exist within the *Drosophila* mushroom body (MB), can potentially account for a very large number of independent, poorly integrated physiological and behavioural phenomena associated with the mushroom body. It considers multiple behavioural roles of MB output neurons beyond attraction and aversion and offers new insight to the how the MB functions in acquisition, consolidation and forgetting of short and long-term memories. They discuss how the motifs and computations discussed would be relevant to other MB functions and altered by known connections, not yet included the simplified model. The manuscript further attempts to show how similar principles could potentially be useful in the mammalian brain. An ambitious and integrative analysis of this sort is sorely needed in the field.

I thank the authors for a very constructive, clear and insightful response to the prior criticism and queries, The manuscript is now hugely improved and can be accepted with no further changes. I think it represents a major contribution to the field. This is a wonderful piece of work that I, at any rate, would recommend to anyone interested in the mushroom body.

*Reviewer #3 (Recommendations for the authors):*

First, I'd like to thank the authors for responding to my concerns/suggestions. At this point, it reads, in my assessment, much better as a result of the many changes. In particular, the newer figures are of high quality and their stated goals much easier to grasp. Also, shifting most of the discussion of the "formal" model in the (old) Results section to the (new) Methods section makes reading flow more intuitively.

Second, the disagreement we had, appears now to be more in terms of naming/labelling of Equation (18) and (30), thus clarifying the rational for the naming of the 2 learning rules (DPR) and (RPE). However, the "RPE" naming for (30) is, in my view, a bit of a stretch, but I am not raising an objection. Just a friendly note to the authors.

I'd like to make a final suggestion that future readers might benefit from. Reviewer 1 raised this issue already and the authors addressed the question. However, in my view, the presentation starting with "we postulate a mathematical formulation …" just above Equation (32), seems a bit circular. While the authors answered the question, in terms of intuitive modeling (Equation (34)), the presentation thread I am referring to is rather formal. The D's in Equations (32), (33) are not explicitly defined; the equations, when added up are consistent with the Equation above line 854. While Equation (34) provides the intuition of the decomposition of the weights into 2 terms, this decomposition is by no means unique. Having said that, we are then confronted with Equations (35) and (36). There is little justification given for the rational of choosing/postulating these two diff. Equations. I presume that the solution for these Equations are the D's. A careful reading seems to suggest that these are delayed differential equations. In math terms, a single delayed diff. Equation is infinite dimensional, and essentially intractable. The following Equations (37)-(39), while consistent with the discussion above, do not help clarify the matter. Which brings one back to Equations (32), (33). Finally, the Methods section has a sizable number of matrices that have seemingly arbitrary entries.

# Reply: Author response

**DOI:** 10.7554/eLife.75611.sa2

[Editors’ note: the authors resubmitted a revised version of the paper for consideration. What follows is the authors’ response to the first round of review.]

While all the reviewers appreciate the ambition and value in integrating diverse sources of data to developing a model of learning, they had some substantial concerns. These are elaborated in their detailed comments, and I provide a distillation of the discussion that the reviewers and I had about the paper. Since it will take considerable further work to address these points, the reviewers and I felt that the paper should be rejected. If the authors wish to resubmit after completely addressing the concerns this would be fine.

We are grateful to the editor and all three reviewers for their enthusiasm for our model, and appreciate the detailed suggestions for how to improve this manuscript. We have taken seriously these comments and have performed substantial revisions of this manuscript to address all concerns raised.

1. The reviewers found the paper a difficult read. Could the authors rewrite to make it accessible to a wide range of readership?

We recognize this as a serious issue and have thoroughly rewritten this manuscript. For example, we have moved the derivation of the dopaminergic plasticity rule to the Methods section, and instead now provide a more intuitive description of the model in the Results section. We also updated the text to concurrently name the mushroom body neurons we believe form the incentive circuit as we describe the specific components of the incentive circuit for ease of comparison. We have updated the behavioural results and methods in order to improve clarity. Several excerpts of this major rewrite are presented below in our responses to each reviewer.

To further improve the accessibility of our manuscript, we have also edited the figures to provide experimental data side-by-side with simulated data to make it easier to compare. Finally, we have changed the way that we illustrate the behaviour of the simulated flies to better show the effects of the plasticity rule and the long-term memory.

2. The formulation of the DLR seems to be a variant of RPE (Reward Prediction Error) learning rules, and hence the conclusions need to be re-evaluated.

Can the authors re-think the basic formulation of DLR starting with Equations (2) and (3)? There should be some experimental tests if the DLR is indeed determined to be different from regular RPE.

We politely disagree that the DLR (now DPR) is a variant of RPE, and have made our explanation of this more clear in the text. For example, “Instead of calculating the error between the reinforcement and its prediction, DPR uses the reinforcement as a driving force to maximise the diversity in the synaptic weights of reinforced experience while reducing it for experiences irrelevant to the reinforcement, which is functionally closer to the information maximisation theory (Bell and Sejnowski, 1995; Lee et al., 1999; Lulham et al., 2011) than the RPE principle.” - lines 103-107.

“Note that this rule [i.e., RPE] allows updates to happen only when the involved KC is active, implying synaptic plasticity even without DAN activation but not without KC activation, which is in contrast with our DPR and recent findings (Berry et al., 2018; Hige et al., 2015) [also in larva (Schleyer et al., 2018, 2020)].” - lines 400-403.

However, we do not exclude the possibility that RPE could be implemented via other mushroom body neurons or connection which are featured in our incentive circuit model.

“… by using the appropriate circuit, i.e., positive MBON-DAN feedback to depressing DANs, our DPR could also have an RPE effect. Although the proposed incentive circuit does not include such connections, it is still possible that they exist.” - lines 484-486.

Finally, we provide a list of testable predictions in Box 1 that includes an experiment to distinguish RPE from DLR, suggested by the combination of the DPR with the incentive circuit.

“By consistently activating one of the LTM MBONs while delivering a specific odour, the LTM MBON should show increased response to that odour even without the use of a reinforcement. This would verify the saturation effect of the DPR and the charging momentum hypothesis. On the other hand, if we observe reduced response rate, this would show that MBON-DAN feedback connection is inhibitory and that RPE is implemented by the circuit.” - lines 644-649.

3. The microcircuits should be better based on experimental data. From our understanding, the data shown in Figures 4H/G, 5B/C/E/F and 6B/C seems to have been obtained by simulations. Would Ca recordings for these figures be feasible? Can there be stronger justification for the connectivity of the proposed incentive circuit?

Those data shown were indeed generated by simulations that highlighted the effects of the different microcircuits combined with the plasticity rule, and Ca recordings for a subset of these experiments already exists and is reproduced in this manuscript. In order to avoid further confusion, experimental and simulated data are now plotted next to each other in the same figure, and we explicitly state in the figure legends whether each subfigure is simulated or experimental data.

We agree that it is critical that our proposed circuit is strongly grounded in experimental data. Our model was designed through close inspection of a comprehensive dataset of DAN and MBON activity during reversal learning in McCurdy et al. (2021), and an examination of the literature regarding anatomical connections between proposed neurons. Hence there is a strong functional and anatomical basis for our circuit. Additionally, our model allows us to make concrete predictions about how neurons would respond in other learning tasks, such as extinction learning and unpaired shock presentation. We found other papers [e.g., Felsenberg et al. (2018), Berry et al. (2018), Ichinoise et al. (2015)] which provide experimental data for some neurons during some of these tasks, which largely aligns with our model predictions. We believe that the few remaining ‘gaps’ in experimental data can be performed by other labs in the future.

3b. The proposed circuit connectivity of the 'incentive circuit' needs to be defined for each MBON because most contemporary work shows that different kinds of memory involved plasticity in different subsets of MBONs. Can the model make specific testable predictions for each subset of MBON?

We include in Table 1 a complete list of known neurons that we propose comprise our circuit connectivity (e.g., MBON-γ1pedc>α/β), how we define it in terms of our model (e.g., s_at_), and the microcircuit in the incentive circuit we propose it is in (e.g., SM). We now also include more discussion of similarities between the proposed properties of our neurons and known properties of memory and plasticity derived from experimental data. For example, we identify MBON-γ1pedc>α/β as an MBON that encodes susceptible memories, which is consistent with how its corresponding DAN, PPL1-γ1pedc, induces a relatively high learning rate and low retention time (Aso and Rubin, 2016).

“Figure 5C-E show the responses of these neurons from experimental data (left) and from our model (right) during aversive conditioning […], which follow a similar pattern. Learning in this circuit is shown by the sharp drop (in both experimental data and model) of the response of MBON-γ1pedc>α/β (Figure 5D) to odour B already from the second trial of the acquisition phase. […] due to our plasticity rule, if the US subsequently occurs without the CS […], the MBON synaptic weights reset due to the recovery effect […]. This is consistent with the high learning rate and low retention time observed in Aso and Rubin. (2016), and it results in a memory that is easily created and erased: a ‘susceptible memory’.” - lines 186-197.

On the other hand, PPL1-γ2α’1 and PAM-β'2a keep the balance between the attraction and avoidance STM, which is also consistent with the more balanced learning rate and retention time in PPL1-γ2α’1 found by Aso and Rubin (2016).

“The ‘charging’ DANs, PAM-β'2a and PPL1-γ2α'1, should be activated directly by reinforcement as well as by the restrained MBONs. This allows for memories to be affected directly by the reinforcement, but also by the expression of the opposite valence memories. The latter feature keeps the balance between the memories by automatically erasing a memory when a memory of the opposite valence starts building up and results in the balanced learning rate and retention time as observed in Aso and Rubin (2016).” - lines 233-238.

We have also provided general predictions regarding specific neurons of the incentive circuit in Box 1.

“MBON-γ2α'1 and MBON-γ5β'2a should exhibit short-term memories, while MBON-α'1 and MBON-β2β'2a long-term memories. MBON-γ1pedc>α/β and MBON-γ4>γ1γ2 should exhibit susceptible memories. Restrained and susceptible MBONs should show more consistent responses across flies. LTM MBONs should have more variable responses because they encode all previous experiences of the animal.” - lines 633-637.

“Blocking the output of charging DANs (i.e., PPL1-γ2α'1 and PAM-β'2a) could reduce the acquisition rate of LTM MBONs, while blocking the output of LTM MBONs would prevent memory consolidation. Blocking the reciprocal connections of the circuit should prevent generalising amongst opposing motivations (unable to make short- or long-term alteration of responses to odours once memories have formed). Blocking the output of forgetting DANs would additionally lead to hyper-saturation of LTMs, which could cause inflexible behaviour.” - lines 650-656.

“Activation of the forgetting DANs should depress the KC-MBON synaptic weights of the restrained and LTM MBONs of the same and opposite valence respectively, and as a result suppress their response to KC activation. Activation of the same DANs should cause increased activity of these MBONs for silenced KCs at the time..” - lines 657-660.

4. Further experimental predictions should be made, based on well-parameterized models of the underlying neurons. Can the authors provide considerably more clarity on which sets of behavioral or physiological data are selected by the authors as targets or tests for specific parts of their model?

We have increased the clarity of the predictions resulting from our model by adding a floating box in the discussion (see Box 1), where we summarise a number of specific predictions (some of them mentioned in the previous comment). We also add a column in Table 1 clarifying whether physiological/anatomical (i.e., using light or electron microscopy) or behavioural/functional (i.e., looking at the responses of postsynaptic neurons while manipulating the pre-synaptic ones) data were used in order to validate the connections of the model.

5. Can the model account for existing data showing overlapping conflicting engrams? Additional experiments and simulations may be needed to ascertain this.

This is an interesting example of sophisticated memory mechanisms in the brain, and both experimental data and our model support this phenomenon. Very recent work (Felsenberg et al., 2018; McCurdy et al., 2021) found that conflicting memories can coexist in the fly brain. For example, MBON-γ1pedc>α/β stores the original aversive memory (odour A = avoidance), and does not change its response to odour A despite multiple subsequent presentations of odour A in the absence of shock. Other MBONs, e.g., MBON-γ5β’2a and MBON-γ2α’1 do in fact change their responses to odour A during extinction/reversal. This phenomenon in part formed the basis for our model, thus our model accounts for these phenomena. While we do not have a complete dataset of all relevant neurons and all learning tasks, our model provides predictions of how these neurons would respond, and this can be verified by experimental labs in the future. We now include this in our results:

“From the summarised synaptic weights shown in Figure 11 —figure supplement 1 [equivalent to engrams], we can see that the susceptible MBONs immediately block the simulated flies from approaching the punishing odours [i.e., original aversive memory], while they allow them to approach the rewarding ones, […]. Susceptible MBONs [i.e., MBON-γ1pedc>α/β and MBON-γ4>γ1γ2] convulsively break the balance between attraction and avoidance created by the restrained and LTM MBONs, also affecting their responses, and allowing STM and as a result LTM formation even without the presence of reinforcement. Figure 11 —figure supplement 1 also show that the restrained MBONs [i.e., MBON-γ5β’2a and MBONγ2α’1] seem to play an important role during the first repeats (up to 5), but then they seem to reduce their influence giving up the control to the LTM MBONs [i.e., MBON-α’1 and MBON-β2β’2a], which seem to increase their influence with time. […] [the different types of MBONs / conflicting engrams] seem to better work when combined, as they complement one another in different stages, e.g., during early or late repeats [of the experiment] and crucial times.” - lines 371-387.

Reviewer #1 (Recommendations for the authors):

This ambitious study builds a model of a proposed key circuit motif in fly behaviour and learning, the Incentive Circuit. The authors examine its implications for a variety of behaviours, and perform a thorough circuit-level mapping of model neuronal activity to recordings. The model uses abstracted model neurons and synaptic signaling, but with careful attention to experimental data at many steps. The mapping to experiments is good, and the model makes far-reaching predictions for animal behaviour.

The development of the model is generally well presented. The learning rule is derived from earlier work (Handler et al) and then the authors transform the terms for ER-ca^2+^ and for cAMP to terms emerging from DA inputs. The model development is especially systematic, building up to the final version step by step with reference to experiments. Importantly, these are mapped to specific sets of experimental observations on the circuit level.

I have mostly comments to clarify or strengthen the presentation.

1. I had a little trouble to envision the two components of D2 and D1. Are they time-varying? Seems to be, see equation 4, where they are presented as D1(t) and D2(t). In other words, do they express D2 and D1 as distinct α functions following spike activity in the DAN? However, in the text and figures it is frequently presented in terms such as D2 > D1 (eg., Figure 3), which looks like a static effect. This was confusing.

We thank the reviewer for this opportunity to clarify our model. D1 (now D_▽_) and D2 (now D_△_) are indeed time-varying, i.e., work as a function with time as a parameter, and we do express them as distinct α functions. We agree with the reviewer that there was potential for confusion, because in the main modelling results for the incentive circuit we use a low time resolution such that these effects can be abstracted to be ‘static’ properties of the influence of specific DANs, even though this is ultimately based on the evidence for two components to the response to DA. The influence (positive or negative ‘dopaminergic factor’) on a particular KC-MBON synapse can still be time-varying as it depends on the activity of all DANs targeting this synapse. We have moved the explanation of the two-component DA response to the methods and now focus on the abstracted concept in the main text. We also updated our description that now makes clear the two terms are time-varying.

“This essentially means that D_▽_(t) and D_△_(t) are expressed as time-varying functions following DAN spike activity. […] we have the fast update with the high peak for D_▽_(t) (0.5 sec for a full update) and a slower update with lower peak for D_△_(t) (1 sec for a full update), …” - lines 873-878.

2. Also in Figure 2A, are we seeing the peak values of ER-Ca or area under curve? Around line 128 it is a hint that it is area under curve, but I am not sure.

It is an approximation of the normalized area under the curve; we refer to it as “the normalised mean change of the synaptic weight” in the manuscript. We have added the paragraph below in order to clarify this now.

“In Figure 16A, we report the normalised mean change of the synaptic weight calculated using the computed ER-ca^2+^ and cAMP levels and the formula below

$\left\langle \Delta W_{k2m}^{\text{ij}}\right\rangle \propto \frac{1}{T}\sum_{t=0}^{T-1}(ER-{\text{Ca}}^{2+}{)}^{\text{ij}}\left(t\right)-(cAMP{)}^{ij}\left(t\right)$” - lines 864-865 and Equation 34.

3. I would have liked to have seen some more mapping to functional experiments in the figures up to Figure 7, where the components of the model are being built up. The authors mention several in the text. Even a qualitative look at the experimental responses would help to strengthen the motivation of the model design.

We agree, and have addressed this point by plotting known experimental data side by side of simulated data for ease of comparison in Figures 5, 6, 7, and 8. We also explicitly state in the text the results of experimental studies and how that data corresponds with what is predicted by our model. E.g.,

“Learning in this circuit is shown by the sharp drop (in both experimental data and model) of the response of MBON-γ1pedc>α/β (Figure 5D) to odour B already from the second trial of the acquisition phase. […] due to our plasticity rule, if the US subsequently occurs without the CS […], the MBON synaptic weights reset due to the recovery effect […]. This is consistent with the high learning rate and low retention time observed in Aso and Rubin. (2016), and it results in a memory that is easily created and erased: a ‘susceptible memory’.” - lines 189-197.

“the experimental data shows a slight drop in the shock response (first paired with odour B, then with odour A) of the DAN, PPL1-γ1pedc, during the whole experiment, although it remains active throughout. We assume this drop may reflect a sensory adaptation to shock but have not included it in our model.” - lines 201-204.

“Interestingly, the [neural] responses communicated by the MB296B_1_ terminal are close to the ones produced by the punishment-encoding charging DAN (see Figure 6C) and the ones of the MB296B_2_ are close to the ones produced by the attraction-driving forgetting DAN (see Figure 8D).” - lines 300-303.

4. The authors then utilize this circuit in an aversive olfactory conditioning paradigm, for which they provide experimental data corresponding to the various neuron types. They then simulate this. This is an outstanding way to validate/test their model. It would be helpful to have the experimental and simulated responses interleaved on the same figure so as to better compare.

We agree and we now plot the experimental and simulated responses side-by-side on the same figure for ease of comparison.

5. I appreciate that it is quite challenging for a simulation to simultaneously replicate properties of several intermediate stages of circuit activity, even more so when the stimulus is not one that the model has been trained on. Could the authors confirm that this is indeed the case, i.e. that the model outcome for figure 9 was obtained only from the parameter tuning earlier in the paper up to Figure 7?

That is correct: the parameters of the model are the same for the whole manuscript. The only parameter that was different was the LTM changing synaptic weight (c->m) in the microcircuits description, and this was just to exaggerate the long-term memory effect and make it more obvious to the reader. As these figures are omitted in our new version of the manuscript, now the parameters are the same for all the results, and we explicitly confirm this in the Methods.

6. It would be useful to perform a statistical evaluation of the fidelity of the model as compared to experiment.

That’s an excellent idea that we now address with Figure 3 —figure supplement 1. We plotted the correlation between behavioural data predicted by our model and experimentally-derived behavioral data from 92 experiments extracted from different studies by Bennett et al. (2021), and found a very strong positive correlation, r = 0.76, p = 2.2 x 10^-18^ (selected neurons) and r = 0.77, p = 2.2 x 10^-19^ (best-fit neurons).

7. The authors then place their model flies in a virtual arena and explore a number of behaviours. Here they contrast their model behaviour with the predictions from a different learning, reward prediction error. I would have liked to have seen in figure 11 an illustration of the correspondence to experimental observations from the literature.

This comment inspired us to perform the additional analysis summarized in Figure 3 —figure supplement 1. In this figure, we compare the correlation between our model and experimental data with the correlation between RPE and experimental data, and found that our model performs better than other models. Pearson’s correlations and p values for DPR, RPE and model presented in Bennett et al., 2021, respectively: r_DPR_ = 0.77, p_DPR_ = 1.65 x 10^-19^; r_RPE_ = 0.58, p_RPE_ = 2.32 x 10^-9^; and r_Bennett_ = 0.68, p_Bennett_ < 10^-4^.

In addition, we provide qualitative evidence of correspondence between our simulated behavior and experimentally-derived behavioural data. For example,

“By looking at the PIs of Figure 11B, we see a strong effect when electric shock is paired with odour A or B, but not very strong otherwise. We also see a smaller π for flies experiencing sugar than the ones that experience electric shock, which is inline with experimental data (Krashes and Waddell, 2011a, b).” - lines 357-360.

Reviewer #2 (Recommendations for the authors):

The manuscript in its current form is built around two main threads. In the first thread, the authors review several results in the literature on associative learning in the mushroom body of the adult fruit fly, and construct an Incentive Circuit (IC) consisting of 6 dopaminergic and 6 mushroom body neurons with specific memory dynamics. They provide a coherent functional view of some of the disparate recent results in associative learning of the mushroom body.

The second thread incorporates a Dopaminergic Learning Rule (DLR) into the IC computational model, providing a computational system for evaluating the learning mechanisms involved.

A weakness here is that the acquisition, forgetting and assimilation of memories qualitatively described in the first thread are not strongly linked with the quantitative IC model described in the second thread.

Conversely, the validation of the IC model circuit, given the noisy data that the authors provide, is only possible in terms of trends, i.e., simple visual inspection. Interpreting the data then is difficult as it does not provide enough constraints for the computational model.

Given the limitations inherent in the validation of the IC from their recorded data, the authors proceed to explore the DLR using behavioral experiments purely based on simulations. This is an effective methodology widely employed in, e.g., robotics. The authors extensively compare the 'learning/navigation' performance of DLR with a variant of reward prediction error (RPE) learning rule and demonstrate a better learning performance. While the comparison may be compelling, we found that underlying the DLR, is the computation of a prediction error, i.e., DLR is a variant of RPE. This calls for a re-evaluation, positioning and clarification of some of the key conclusions regarding why the DLR is effective in associative learning tasks.

Substantive concerns

1. l.128 The section 'Mushroom Body Microcircuits' makes good first reading. However, most of the key statements could further benefit from more extensive quantitative backing as hinted at in Figures 4, 5 and 6 (see also my comment below). Since these microcircuits are simpler than the IC, my expectation is that they could provide better intuition regarding their function.

This is an excellent point. We now plot the quantitative experimental data side-by-side with the simulated data for ease of comparison. We also include the corresponding neuron name with the model neuron’s name, for better intuition, as the reviewer suggested.

“Learning in this circuit is shown by the sharp drop (in both experimental data and model) of the response of MBON-γ1pedc>α/β (Figure 5D) to odour B already from the second trial of the acquisition phase. […] due to our plasticity rule, if the US subsequently occurs without the CS […], the MBON synaptic weights reset due to the recovery effect […]. This is consistent with the high learning rate and low retention time observed in Aso and Rubin. (2016), and it results in a memory that is easily created and erased: a ‘susceptible memory’.” - lines 189-197.

“the experimental data shows a slight drop in the shock response (first paired with odour B, then with odour A) of the DAN, PPL1-γ1pedc, during the whole experiment, although it remains active throughout. We assume this drop may reflect a sensory adaptation to shock but have not included it in our model.” - lines 201-204.

“Interestingly, the [neural] responses communicated by the MB296B_1_ terminal are close to the ones produced by the punishment-encoding charging DAN (see Figure 6C) and the ones of the MB296B_2_ are close to the ones produced by the attraction-driving forgetting DAN (see Figure 8D).” - lines 300-303.

2. Figures 4F and 4G are rather difficult to understand/parse. More caption details, choice of different colors, would help.

Same comment regarding Figures 5B, 5C, 5E and 5F, and 6B, 6C.

Based on this comment and similar sentiments expressed by other reviewers, we have now removed these figures. Instead, we made new figures (i.e., Figures 5, 6, 7, and 8) which plot known experimental data side by side of simulated data for ease of comparison.

3. While Figure 8 is to be commended, the data is rather noisy and, in my view, despite the best intentions, rather difficult to understand/evaluate. As the authors argue in l.312, 'we computationally modelled the incentive circuit in order to demonstrate all the properties we described before and compare the reconstructed activities to the ones of Figure 8C'. However, a comparison by simple visual inspection is rather unconvincing. The need for introducing a distance measure is in order.

Although we do not incorporate a distance measure, we have made two major changes to address this issue: First, we plot the experimental data next to the simulated data so that readers can perform visual inspection more easily. Second, we now provide explicit descriptions of the level of similarity between recorded and simulated data for each neuron. Overall, there is a large degree of overlap for the majority of neurons e.g., PPL1-γ1pedc, both PPL1-γ2α’1, PAM-β’2, MBON-γ1>α/β, MBON-γ5β’2a, MBON-γ2α΄1 and MBON-β2β’2. However, it is interesting that some neurons, e.g., the MBON-α’1, do not have as good of a fit. We discuss this in the text and provide possible reasons for why these neurons in particular do not fit as well.

“Figure 5C-E show the responses of these neurons from experimental data (left) and from our model (right) during aversive conditioning (the paradigm shown in Figure 4), which seem to follow similar patterns.” - lines 186-188.

“However, these trends are not evident in the experimental data as illustrated in Figure 7D (left). We suggest this is because responses of long-term memory neurons depend on the overall experience of the animal and are thus hard to predict during one experiment. For example, it could be the case that the animal has already built some long-term avoidance memory for odour A, such that its presentation without reinforcement in our experiment continues its learning momentum leading to the observed increasing response.” - lines 266-272.

4. I found 'modeling behavior', as presented in the current version of the manuscript, to be quite effective. However, I'd like to note that in the process, the authors changed the underlying PN activity model. This requires, given that the rest of the paper is based on a binary odor model of the PN activity (see the discussion preceding Equation (6)), some careful/detailed assessment of its implications.

This is a valid point. For consistency we reran our simulations in Figure 11 using the same PN activity parameters (binary, using a threshold on odour intensity) as those used in the earlier figures. These new results are comparable to the previous version, and still support our conclusions. We have now included the specifics of this model here:

“Note that PN responses depend only on the fact that an odour has been detected or not and it is not proportional to the detected intensity.” - lines 812-813.

5. Finally, the authors propose to compare their DLR with a variant of RPE. Here a major conceptual problem arises. The authors argue that DLR is a fundamentally different learning rule from RPE. They state in l.462 that 'The idea behind RPE is that the fly learns to predict how rewarding or punishing a stimulus is by altering its prediction when this does not match the actual reward or punishment experience'. This can be adapted to the mushroom body circuit by assuming that the MBON output provides a prediction of DAN activity. But this is exactly what Equation (18) states. The differential equation (18) describing the gradient of the DAN activity is equal to sum of the weighted shock delivery ('transform' in l.750) and the weighted MBON activity (l.755). The sum is just the prediction error between the two terms. Consequently, since the DLR is, in view of this reviewer, a variant of RPE, a comparison with another RPE is of little interest. A substantial re-write of the paper starting with the section on the Incentive Circuit (l. 257) is in order.

We believe we all agree that our DPR (with the simplest circuit implementation as shown in Figure 2) is definitely not a variant of the RPE.

“Instead of calculating the error between the reinforcement and its prediction, DPR uses the reinforcement as a driving force to maximise the diversity in the synaptic weights of reinforced experience while reducing it for experiences irrelevant to the reinforcement, which is functionally closer to the information maximisation theory (Bell et al., 1995; Lee et al., 1999; Lulham et al., 2011) than the RPE principle.” - lines 103-107.

The reviewer is right that (in the incentive circuit) DAN responses are indeed calculated based on the weighted US plus the weighted MBON activity. However, as the DAN and US responses are always positive numbers, and the synaptic weights are also positive, this is not (in general) the calculation of an error. An exception is the s->d connection, which is inhibiting. However, even for this case, although this term could be interpreted as error calculation, only the positive part of the DAN activity is used in the learning rule (see Equation 22) which means that the MBON activity (indirectly passed through the DAN activity) only controls the magnitude and not the ‘direction’ of change for the synaptic weight. It is thus clearly different to RPE methods that control both the magnitude and direction of change based on the error computed between the reinforcement and its ‘prediction’.

“Consequently, the model data shows a positive feedback effect: the DAN causes depression of the MBON response to odour, reducing inhibition of the DAN, which increases its response, causing even further depression in the MBON. Note this is opposite to the expected effects of reward prediction error.” - lines 205-208.

That said, we mention in our Discussion the possibility that RPE could be implemented by other neurons or connections of the mushroom body (not included in the incentive circuit):

“However, although the evidence for MBON-DAN feedback connections is well-grounded, it is less clear that they are consistently opposing. For example, in the microcircuits we have described, based on neurophysiological evidence, some DANs that depress synaptic weights receive inhibitory feedback from MBONs (Pavlowsky et al., 2018) and some DANs that potentiate synaptic weights receive excitatory feedback from DANs (Ichinose et al., 2015). As we have shown, the DPR is able to operate with this variety of MBON-DAN connections. Note that, by using the appropriate circuit, i.e., positive MBON-DAN feedback to depressing DANs, our DPR could also have an RPE effect. Although the proposed incentive circuit does not include such connections, it is still possible that they exist.” - lines 478-486.

6. l.765: "The above matrices summarise the excitatory (positive) and inhibitory (negative) connections between MBONs and DANs or other MBONs. The magnitude of the weights was hand-tuned in order to get the desired result." This 'hand-tuning" appears, to me, to be a 'construction' of the prediction error on the right hand side of Equation (18). Some details might help clarify to what extent the hand-tuning is based on the assumptions of the binary model of the 2 odors at the PN level. I presume that the generality of the model alluded to in l.743 stating that 'that the number of neurons we are using for PNs and KCs is not very important and we could use any combination of PN and KC populations' breaks down and the hand-tuning needs to be repeated every time the number of neurons is changed.

We have now made clearer in the methods that the ‘hand-tuning’ of weight magnitude does not permit alteration of the sign of the weights, and it does not result in effective construction of prediction error, as detailed in our previous answer. The tuning is used to create a better matching between the recorded and reconstructed responses in Figures 5-9, and so as to keep the balance of memories in the circuit, e.g., the MAM forgetting should be equally weighted to the LTM charging so that we erase from the STM the same amount as we store in the LTM, and it is independent to the PN activity pattern. The weights are not further changed for the remainder of the results. Finally, by hand-tuning we want to emphasise that we haven’t used any automatic, unconstrained method to calculate the weights in order to fit the data better. We have now edited the text to reflect this:

“… we define these parameters and some properties of our computational model, which are not a result of unconstrained optimisation and are consistent throughout all our experiments.” - lines 705-706.

“The sign of the weights was fixed but the magnitude of the weights was hand-tuned in order to get the desired result, given the constraint that equivalent types of connections should be same weight (e.g., in the reciprocal microcircuits). The magnitude of the synaptic weights specify the effective strength of each of the described microcircuits in the overall circuit.” - lines 743-746.

Reviewer #3 (Recommendations for the authors):

The authors propose an original dopaminergic learning rule, which, when implemented in simple neural circuit motifs shown to exist within the *Drosophila* mushroom body (MB) , can potentially account for a very large number of independent, poorly integrated physiological and behavioural phenomena associated with the mushroom body. It considers multiple behavioural roles of MB output neurons beyond attraction and aversion and offers new insight to the how the MB functions in acquisition, consolidation and forgetting of memories. The manuscript further attempts to show how similar principles could potentially be useful in the mammalian brain. An ambitious and integrative analysis of this sort is sorely needed in the field.

The paper has obviously involved very broad and deep consideration of the MB connectome as well as genetic, physiological and behavioural studies of the roles of the different classes of Kenyon cells, MBONs and DANs that innervate the mushroom body. It is original and ambitious and potentially very valuable to the field.

My major reservation is that the manuscript is very difficult to read and evaluate by anyone who is not a *Drosophila* mushroom body aficionado. I consider myself an interested reader and one who keeps broad track of the field, but found the need to read and evaluate far too many papers cited by the authors to decide how well phenomena the authors attempt to model have been demonstrated and how well assumptions made by the authors are justified by data.

1. E.g. I was stymied even at figure 1, where mutual inhibition between MBONs is indicated and it took me considerable (and eventually futile) effort to look into where and how well this has been established.

In Figure 1 we meant to demonstrate that MBON-to-MBON connections exist in the mushroom bodies, and it was not our intention to suggest mutual inhibitory connections. We have changed the lines of these connections to dashed, so that they look different from the rest of the connection. We have also updated the caption of Figure 1 to make this clear.

“These circuits include some direct (but not mutual) MBON-MBON connections (dashed inhibitory connections).” – Figure 1.

2. To make the work more accessible at least to this moderately educated reviewer, I fear that a major re-rewrite will be required. I would suggest that for each section – exactly has been shown be clearly enumerated, with enough detail provided for the reader to judge the strength of these data. The justification and support for three types of MBONs and their incentive should also be particularly clearly indicated.

We have undertaken a major rewriting and we hope the manuscript is now easier to process, including for those less familiar with the *Drosophila* mushroom body. This includes more explicit connection of each part of the circuit construction to the relevant data.

3. Moreover, while the authors are correct to point out the limitations of current models based on dopamine prediction-error, I do wonder if there is room for prediction error to also contribute meaningfully within the framework proposed in this paper.

Indeed, we believe that there is still room for RPE in the fly brain, that could also be implemented by our DPR given specific circuitry as we discuss in our text:

“… this rule [i.e., DPR], in combination with some specific types of circuits, can result in prediction of reinforcements, …” - lines 107-108.

“… by using the appropriate circuit, i.e., positive MBON-DAN feedback to depressing DANs, our DPR could also have an RPE effect. Although the proposed incentive circuit does not include such connections, it is still possible that they exist.” - lines 484-486.

However, we also believe it is not a general (or necessary) property of plasticity in the mushroom body, as we illustrate in our incentive circuit.

I apologise for the not having a list of specific issues for the authors to address, because I found the basis to be so difficult to explore but here is some general feedback.

4. It is nice that and the dynamics of neural responses obtained with the model correspond closely with ones reported in previous studies (although there are exceptions, some nicely highlighted by the authors).

We thank the reviewer and we are happy that they see the value of our work.

5. There should be deeper engagement with signalling mechanisms that differentiate the two types of dopamine receptors. I found the assumptions regarding their differences to be useful for the modelling of different effects of reinforcement before or after sensory experience (Ruta Cell 2019), but quite superficial in terms of providing hypothesis for how the receptors may differ in terms of mechanism of action.

D_1_ and D_2_ (now D_▽_ and D_△_, respectively) are not necessarily meant to be DopR1 and DopR2 responses.

These are 2 abstract terms/components of the dopaminergic signal that interact in the synapse and might be related to DopR1 and DopR2, but they are not the same. We hope that this is clearer now in our text:

“… where D^j^_▽_(t) and D^j^_△_(t) are the depression and potentiation components of the DA respectively [assumed to correspond to DopR1 and DopR2 receptors (Handler et al., 2019), or potentially to involve cotransmitters released by the DAN such as Nitric Oxide (Aso et al., 2019)].” - lines 845-847.

6. ON the same note, specific experimental predictions of the model could also be clearly indicated at the end of each section.

We have now added a floating box (Box 1) with specific experimental predictions of the model. Some examples of these predictions include: (a) the roles of the different DANs and MBONs in the memory dynamics of fruit flies, (b) how the activity of specific neurons would be affected when manipulating the activity of specific neurons in the mushroom body and (c) what are the effects of manipulating the neurons in different conditioning types (e.g., first-order, second-order and unpaired).

7. While the authors admittedly designed informative and clear figures, and their Table 1 points the reader to papers that report relevant neural connections and neuronal functions, this is not enough. Data in support of each assumption should be clearly and specifically mentioned and hypotheses connections also clearly stated. After considerable effort, I still could find no evidence for the existence of inhibitory connections between MBONγ4 and MBONγ2 (which is not to say that none exist – but surely it is the authors job to clarify this).

We have updated Table 1 to make more clear what information about neural connections is known versus hypothesized. For each connection, we denote whether its anatomical connection (using light microscopy or electron microscopy) or functional connection (i.e. whether activating the presynaptic neuron leads to an excitatory or inhibitory response in the postsynaptic neuron, and/or the neurotransmitter released by the presynaptic neuron) is known.

Regarding the inhibitory connection between MBON-4 and MBON-2, we assume that the reviewer refers to the depressing dopaminergic effect of PAM-04 (i.e., PAM-β2β’2a, f_av_) to the KC-MBON synapses of MBON-02 (i.e., MBON-β2β’2a, m_at_) in the reciprocal LTM microcircuit. We based our assumption that this effect exists on Aso et al. (2014) and Li et al. (2020), who support that specific MBONs that extend their dendrites in compartments where specific DANs terminate their axons are affected by dopamine emitted by them. However, in most cases it is unclear whether the effect of this dopamine is potentiating or depressing, which we try to infer by using the data from McCurdy et al. (2021). Exceptions are the microcircuits described by Pavlowsky et al. (2018), Felsenberg et al. (2018), McCurdy et al. (2021) and Ichinose et al. (2015), who experimentally show the sign of dopamine effect onto the target synapses of the specific MBONs, which we take into account and use them as is in the model. The rest of the effects are postulated either by the symmetry of the circuit or from logic of what the desired function is.

8. The authors should also try to account for the discovery of parallel, independent memory traces (like appetitive LTM formation towards the CS- in classic LTM aversive training paradigms).

We agree with the reviewer that this is an important phenomenon and it should be addressed. There are multiple ways that parallel memories are built in our model. First, referring to memories of the same odour (transmitted by the same KC population), and second, to individual odours (transmitted by different KC populations). We think that it is now clear in our manuscript that independent memory traces are formed in the susceptible MBONs (as the activity of the one does not depend on the other – not connected in any way), while STM and LTM MBONs store dependent memories (as they are connected reciprocally and build dependencies). We mention this here:

“The restrained MBONs activate their respective ‘charging’ DANs, which start to potentiate the ‘LTM’ MBONs of same valence, while also depressing the response (to KC input) of the restrained MBON of opposite valence.” - lines 159-161.

“… the susceptible MBONs immediately block the simulated flies from approaching the punishing odours, while they allow them approach the rewarding ones, […]. This is partially because of the lack of reciprocal connections between the opposing susceptible MBONs, and it can be verified through the appetitive conditioning, […]. Susceptible MBONs convulsively break the balance between attraction and avoidance created by the restrained and LTM MBONs, …” - lines 372-378.

On the other hand, memories associated to different odour identities are formed in parallel through the different populations of KCs (i.e., their connections to MBONs). Although these memories are in principle independent, they can be dependent if the populations of two odours are overlapping.

“… our results show that (in time) the simulated flies seem to develop some prior knowledge about both odours when experienced at least one of them with reinforcement (see Figure 11B and Figure 11 —figure supplement 2A), which we suggest is because of their overlapping KCs associated with both odours.” - lines 363-366.

9. Does the dopaminergic learning rule explain the differences in dynamics and memory strength between appetitive and aversive memories? These two types of memory involved different molecular components and display different learning rules (stronger short-term aversive memories and longer-lasting appetitive memories requiring less training)? This should perhaps be clarified, particularly since KC output appears dispensable for aversive learning (acquisition) but potentially necessary for the acquisition of appetitive memories (Pribbenow et al., 2021).

That’s an excellent question! Indeed, the DPR produced similar findings in our simulations of behavioural experiments, in terms of dynamics and memory strength between appetitive and aversive memories. Our (simulated) behavioural experiments show that this difference in dynamics and memory strength between appetitive and aversive memories is a result of the behaviour itself and has nothing to do with the plasticity rule or the circuit. Specifically, although the DPR and IC are characterized by complete symmetry, the fact that flies attracted by an odour tend to spend more time experiencing this odour, while flies avoiding an odour tend to spend less time experiencing it, actually produces this difference in the learning outcome. So we predict that the mechanism that handles both cases is exactly the same, but a more naturalistic condition is needed in order to see this effect. This is now highlighted in our manuscript:

“We […] see a smaller π for flies experiencing sugar than the ones that experience electric shock, which is inline with experimental data (Krashes and Waddell, 2011a,b). When shock is paired with both odours we expect that the simulated flies will try to minimise the time spent exposed to any of them […]. In contrast, simulated flies seem to increase the time spend in both odours when paired with sugar with a slight preference towards the reinforced odour.” - lines 358-363.

10. I found the easy assumption that forgetting involves erasure to be troubling. Perhaps this happens sometimes. But many apparently "forgotten" memories are never erased, simply not reactivated for multiple reasons. Intellectually this point needs to be acknowledged.

We thank the reviewer for the opportunity to refine our wording. As the reviewer points out, there are multiple neural mechanisms that could lead to the behavioral manifestation of a “forgotten” memory, e.g., that the fly no longer avoids an odour previously paired with aversive stimuli. In some instances, the original aversive memory undergoes decay over time (e.g., susceptible MBONs during extinction and unpaired learning).

“… due to our plasticity rule, if the US subsequently occurs without the CS (see unpaired phase in the model, for which we do not have fly data), the MBON synaptic weights reset due to the recovery effect…” - lines 193-195.

In some cases (e.g., restrained and LTM MBONs), it remains intact but competes with a new parallel memory formed when the odour is presented without electric shock, as in extinction or reversal learning.

“The response of MBON-γ5β'2a (Figure 5E) can be observed to have the opposite pattern [to the MBONγ1pedc>α/β], i.e., it starts to respond to odour B from the second trial of acquisition as it is no longer ‘restrained’. Note however that the response it expresses, when the restraint is removed, also depends on its own synaptic weights for KC input, which as we will see, may be affected by other elements in the incentive circuit.” - lines 197-201.

In our model, although memories in the susceptible and restrained MBONs are constantly updated, LTM MBONs integrate these memories and save them for a long time through saturation.

“Figure 7D (right) demonstrates the charging of the avoidance-driving LTM MBON during the acquisition (for odour B) and its continued increase during the forgetting phases.” - lines 265-266.

However, even when the memories in the LTM MBONs are weakened (e.g., due to the reciprocal LTM connections), we suggest that they are further assimilated by higher level LTMs in the vertical lobes of the MB, but this is not part of our circuit and needs further investigation.

“… we predict that the function of the cingulate cortex is represented by the α/β MBONs, encoding the ‘emotions’ of the animal towards reinforced stimuli, potentially controlling more sophisticated decision making.” - lines 691-694.

[Editors’ note: what follows is the authors’ response to the second round of review.]

The manuscript has been improved but there are some remaining issues that need to be addressed, as outlined below:

1. Could the authors compare their simulated/predicted behavior with some quantitative or semi-quantitative measures of experimental behavior?

We recognise the need for quantitative comparison between our results and the literature. For this reason, in our behavioural Results section, we highlight more clearly the results reported in our Figure 3 —figure supplement 1, showing (using distillation to a common metric) a high correlation between the behaviour produced by our model and data from 92 classical conditioning experiments.

“Following this approach and using the summarised data collected by Bennett et al. (2021), we have tested the performance of our model in 92 olfactory classical conditioning intervention experiments from 14 studies (Felsenberg et al., 2017; Perisse et al., 2016; Aso and Rubin, 2016; Yamagata et al., 2016; Ichinose et al., 2015; Huetteroth et al., 2015; Owald et al., 2015; Aso et al., 2014b; Lin et al., 2014; Plaçais et al., 2013; Burke et al., 2012; Liu et al., 2012; Aso et al., 2010; Claridge-Chang et al., 2009), i.e., the observed effects on fly learning of silencing or activating specific neurons, including positive and negative reinforcements. The Δf predicted from the incentive circuit correlated with the one reported from the actual experiments with correlation coefficient r=0.76, p=2.2 x 10^-18^ (Figure 3 —figure supplement 1).” – lines 333-342.

2. Can the authors elaborate on their mapping of ER-CA and cAMP in the model with the cited data? This relates to point 4 from Reviewer 1.

We also noticed that the individual traces of ER-Ca and cAMP (Figure 16B) do not match exactly the data from Handler et al. (2019). However, Figure 16A (and B – ΔW, i.e., black line) shows that their effect (combination of the two) is very similar to the one presented by the original paper (Pearson correlation: r=0.98, p=3.9 10^-4^). To allow direct comparison, we now plot the modelled ER-Ca and cAMP on the top of the data from Handler et al. (2019) in Figures 16A and B (grey lines). Note that we do not claim to model the exact ER-Ca and cAMP levels and we hope that this is now clear in the text.

“Figure 16 shows the ER-Ca^2+^ and cAMP levels during forward and backward conditioning for a depressing DAN […], which are comparable to the data shown in Handler et al., (2019) (also Figure 16 – shown in grey). Note that here we are more interested in the overall effects of learning shown in Figure 16A rather than the detailed responses of Figure 16B.” – lines 869-873.

3. Can the authors do some parameter sensitivity analysis as suggested by the reviewers?

We added an extensive search/analysis on the timing parameters (i.e., τ_short_ and τ_long_) of the plasticity rule where we compare the correlation between the data and the effect of our equation (i.e., Figure 16A). We have now added Figure 16 —figure supplement 2 showing the results of this analysis.

“In Figure 16, where we are interested in more detailed dynamics of the plasticity rule, and the sampling frequency is high, i.e., 100 Hz, we use τ_short_ = 60 and τ_long_ = 104, which we choose after a parameter exploration available in Figure 16 —figure supplement 2” – lines 893-895.

Regarding the circuit, the synaptic strengths are hand tuned in order to make the plots in Figures 5-8 (at least visually) match. A parameter analysis (in the same way that we did it for the plasticity rule, i.e., comparing the reproduced responses to the data using a standard measure, e.g., Pearson correlation coefficient) is less effective and harder to make for these parameters, as each connection affects the responses of many neurons in the circuit. Instead, we have created Figure 14 —figure supplements 1, 2 and 3, which show how the responses of the neurons in the circuit alter by changing one parameter at a time.

“Figure 14 —figure supplement 1, Figure 14 —figure supplement 2 and Figure 14 —figure supplement 3 show how each of these parameters affect the responses of the neurons in the incentive circuit.” – lines 769-771.

In addition, the reviewers had a few points for the authors to expand upon in the revision, and a number of useful suggestions to improve clarity.

Reviewer #1 (Recommendations for the authors):

This is an ambitious but also highly complicated modeling study that seeks to account for a wide range of fly learning behaviour in terms of underlying learning rules and circuitry.

The strengths of the study are its ambition, detail and substantial attention to experimental inputs. In principle it builds up a large and testable conceptual framework for understanding many aspects of learning. Its weaknesses, which are readily fixed, are 1. that the study misses opportunities to better compare model to experiments. and 2, that the study doesn't do a systematic parameter and model exploration to see how robust are the properties.

With these additions the study would be strong and of value to the field in laying out a template for further investigation. The authors posit that this framework could also apply to other organisms.

General points:

1. This is an ambitious but also highly complicated modeling study that seeks to account for a wide range of fly learning behaviour in terms of underlying learning rules and circuitry. The authors have made substantial improvements to the clarity of the presentation, particularly with regards to comparison of experimental and simulated data.

I would have liked to see similar comparison for two more features: the behaviour, and the crucial learning rule section, as I comment below. I note that a similar request was made in an earlier review.

The reviewer has expanded on this issue in points 3 and 4 and we respond there.

2. The other big thing I would have liked to see is an exploration of parameter sensitivity. This is needed both because of model complexity and because of the not-perfect match between model and input data. No model is perfect, but the confidence in a model is much improved if one can see that it still 'works' even when the numbers (and other assumptions) shift around a bit.

The reviewer has expanded on this issue in point 5 and we respond there.

3. Behaviour: The authors have made the interesting and potentially powerful step of linking their model to measurable behaviour. But they miss the opportunity to put the outcomes (experiment and model) side by side. Even a semi-quantitative distillation to some common metric for displaying and comparing the experimental and model properties would have been valuable.

We recognise the need for quantitative comparison between our results and the literature. For this reason, in our behavioural Results section, we highlight more clearly the results reported in our Figure 3 —figure supplement 1, which shows (using distillation to a common metric) a high correlation between the behaviour produced by our model and data from 92 classical conditioning experiments.

“Following this approach and using the summarised data collected by Bennett et al. (2021), we have tested the performance of our model in 92 olfactory classical conditioning intervention experiments from 14 studies (Felsenberg et al., 2017; Perisse et al., 2016; Aso and Rubin, 2016; Yamagata et al., 2016; Ichinose et al., 2015; Huetteroth et al., 2015; Owald et al., 2015; Aso et al., 2014b; Lin et al., 2014; Plaçais et al., 2013; Burke et al., 2012; Liu et al., 2012; Aso et al., 2010; Claridge-Chang et al., 2009), i.e., the observed effects on fly learning of silencing or activating specific neurons, including positive and negative reinforcements. The Δf predicted from the incentive circuit correlated with the one reported from the actual experiments with correlation coefficient r=0.76, p=2.2 x 10^-18^ (Figure 3 —figure supplement 1).” – lines 333-342.

4. Figure 16: Details of ER-CA and cAMP in the model don't match data. The form of the pairing for ER Ca is inconsistent with the data of Handler et al., particularly when CS precedes US by a large interval. Handler et al. show no response for forward pairing even several seconds after the last stimulus. Also, the time-course of ER response for the backward pairing case is inconsistent. In the Handler data (Figure 6) the ER signal remains low (i.e, very different from baseline) well past 5 seconds, whereas in Figure 16 the signal returns to baseline within 5s. I am also concerned that there doesn't seem to be experimental support for the reduced cAMP signal at very small overlap intervals. Indeed, the Handler data suggests that there is a large signal at the 0.5s and -1.2s points. Figure 16 shows that the model assumes a low and brief signal at -1.2s. I would have appreciated having the experimental data from Handler and others illustrated here in the same figure, just to see how well the model forms behave. It would save the reader the step of going to look up another paper and tracking down appropriate figure panels.

We agree with the reviewer that the individual traces of ER-Ca and cAMP do not match exactly the data from Handler et al. (2019). On the other hand, Figure 16A (and B – ΔW, i.e., black line) shows that their effect (combination of the two) is very similar to the one presented by the original paper (r=0.98, p=3.9 10^-4^). The authors of Handler et al. (2019) have kindly provided the data from their figures, which allows us to report the Pearson correlation coefficient and also explore the timing parameters (requested in a different point). Thus, we now plot the modeled ER-Ca and cAMP on the top of the data from Handler et al. (2019) in Figures 16A and B (grey lines) as the reviewer suggested. Note that we do not claim to model the exact ER-Ca and cAMP levels and we hope that this is now clear in the text.

“Figure 16 shows the ER-Ca^2+^ and cAMP levels during forward and backward conditioning for a depressing DAN […], which are comparable to the data shown in Handler et al., (2019) (also Figure 16 – shown in grey). Note that here we are more interested in the overall effects of learning shown in Figure 16A rather than the detailed responses of Figure 16B.” – lines 869-873.

5. As one example of a useful parameter sensitivity analysis: The form of the deltaWij seems rather crucial to the model, so I'm homing in on this. It is a difference of two values which are themselves clearly the difference of opposing signals. It would therefore be valuable to show that relaxation of these tight timing requirements does not upset the learning rule and subsequent behaviour. It would be useful to see similar sensitivity analyses for other key parts of the model.

We agree with the reviewer that it would be very interesting to explore all the parameters of the model. This way we could show how sensitive the predictions of the model are in the selection of its parameters. Given that we now have the Handler et al. (2019) data, we added an extensive search/analysis on the timing parameters (i.e., τ_short_ and τ_long_) of the plasticity rule where we compare the correlation between the data and the effect of our equation (i.e., Figure 16A). We have now added Figure 16 —figure supplement 2 showing the results of this analysis.

“In Figure 16, where we are interested in more detailed dynamics of the plasticity rule, and the sampling frequency is high, i.e., 100 Hz, we use τ_short_ = 60 and τ_long_ = 104, which we choose after a parameter exploration available in Figure 16 —figure supplement 2” – lines 893-895.

Regarding the circuit, an extensive parameter search is much harder to make. The parameters of the circuit include the strength of a connection between MBONs and post-synaptic targets (e.g., other MBONs or DANs), the modulatory strength of the different types of DANs onto target KC>MBON synapses and the biases (i.e., resting activity) of the neurons. In our approach, the synaptic strengths are hand tuned in order to make the plots in Figures 5-8 (at least visually) match. A parameter analysis (in the same way that we did it for the plasticity rule, i.e., comparing the reproduced responses to the data using a standard measure, e.g., Pearson correlation coefficient) is less effective and harder to make for these parameters, as each connection affects the responses of many neurons in the circuit. Instead, we have created Figure 14 —figure supplements 1, 2 and 3, which show how the responses of the neurons in the circuit alter by changing one parameter at a time.

“Figure 14 —figure supplement 1, Figure 14 —figure supplement 2 and Figure 14 —figure supplement 3 show how each of these parameters affect the responses of the neurons in the incentive circuit.” – lines 769-771.

Clarifications:

6. pg 28: 3 lines from bottom.

Do the authors mean "activity of the ith presynaptic KC? 'Target' sounds like it is postsynaptic.

The reviewer is right and we have changed this as suggested.

“the activity of the (i^th^) pre-synaptic KC” – page 29, 3 lines from bottom.

7. Equation 30 onward.

w_rest: Is this a global parameter for all synapses?

w_rest: The way it is used in the equation looks more like a_rest, the resting activity of the synapse. Sorry to be pedantic, but the units of weight and rate don't match.

This gets further mixed in the equation between lines 853 and 854 where the authors add ki and Wij. Maybe ki is scaled somehow to weights?

*w*_*rest*_ is a global parameter that corresponds to the default weight of a variable synapse and the weight to which it tends to return. It could in principle differ for different synapses but for simplicity we here assume that all the KC>MBON synapses (the only variable synapses in our model) should have the same resting weight. We appreciate the issue that ‘synapse weight’ and ‘neuron activity (firing rate)’ are not intrinsically the same units but have indeed implicitly scaled the latter (*k*^*i*^) to allow it to be compared to the current weight in the relevant weight-change equation. In general, it is common to all neural plasticity rules to assume there is some direct conversion from activity levels to weight changes.

8. Figure 5 and later: The responses, both experimental and model, are shown as an up-down oscillation. I assume that the up states are measurements during the training, and down is measurement half a day later. But this is hard to see from the text or legends, and I had to go down to the last section in the methods to see that this seems to be described as on-shock and off-shock values. It is confusing and should be mentioned in the figure legends and accompanying text.

We thank the reviewer for noticing this. The oscillations in the responses are due to the on- and off-shock values, not of different days but of consecutive time-windows (expose the animal to the odour only – i.e., off-shock – before introducing shock along with the odour – i.e., on-shock). We have now updated the captions of Figures 5-8 (and figure supplements) to clarify that.

“For each trial we report two consecutive time-steps: the off-shock (i.e., odour only) followed by the onshock (i.e., paired odour and shock) when available (i.e., odour B in acquisition and odour A in reversal phase) otherwise a second off-shock time-step (i.e., all the other phases).” – Figure 5-9 and the respective Figure supplements.

Reviewer #3 (Recommendations for the authors):

First, I'd like to thank the authors for responding to my concerns/suggestions. At this point, it reads, in my assessment, much better as a result of the many changes. In particular, the newer figures are of high quality and their stated goals much easier to grasp. Also, shifting most of the discussion of the "formal" model in the (old) Results section to the (new) Methods section makes reading flow more intuitively.

Second, the disagreement we had, appears now to be more in terms of naming/labeling.of Equation (18) and (30), thus clarifying the rational for the naming of the 2 learning rules (DPR) and (RPE). However, the "RPE" naming for (30) is, in my view, a bit of a stretch, but I am not raising an objection. Just a friendly note to the authors.

I'd like to make a final suggestion that future readers might benefit from. Reviewer 1 raised this issue already and the authors addressed the question. However, in my view, the presentation starting with "we postulate a mathematical formulation …" just above Equation (32), seems a bit circular. While the authors answered the question, in terms of intuitive modeling (Equation (34)), the presentation thread I am referring to is rather formal. The D's in Equations (32), (33) are not explicitly defined; the equations, when added up are consistent with the Equation above line 854.

We do define D’s as the depression and potentiation components of the DA, assumed to correspond to DopR1 and DopR2 receptors or potentially to involve co-transmitters released by the DAN such as Nitric Oxide.

“where D^j^_▽_ (t) and D^j^_△_(t) are the depression and potentiation components of the DA respectively [assumed to correspond to DopR1 and DopR2 receptors (Handler et al., 2019), or potentially to involve cotransmitters released by the DAN such as Nitric Oxide (Aso et al., 2019)].” – lines 863-865.

While Equation (34) provides the intuition of the decomposition of the weights into 2 terms, this decomposition is by no means unique. Having said that, we are then confronted with Equations (35) and (36). There is little justification given for the rational of choosing/postulating these two diff. Equations. I presume that the solution for these Equations are the D's. A careful reading seems to suggest that these are delayed differential equations. In math terms, a single delayed diff. Equation is infinite dimensional, and essentially intractable. The following Equations (37)-(39), while consistent with the discussion above, do not help clarify the matter. Which brings one back to Equations (32), (33).

We are grateful that the reviewer had such a close look to our equations which lead us to have a closer look as well. We came up with Equations (35) and (36) as a simple model of the shape of rise and decay responses to DA release. However, thanks to the reviewer’s comments, we realised that there is a mistake in the differential equations and the τ_short_ and τ_long_ parameters in our equation. The correct values are τ_short_ = 1 and τ_long_ = +∞_._ Equations 35 and 36 have been amended accordingly.

Note that now we remove the time as a parameter in these equations so that it becomes less confusing and closer to standard notation. The above differential equations and parameters can be used to generate the plasticity rule as in Equations (37)-(39):

$$\frac{{\text{dD}}_{\nabla }}{\text{dt}}+D_{\nabla }=-d^{T}∙W_{d2km}^{-}\;D_{\nabla }\left(t\right)=-d^{T}(t)∙W_{d2km}^{-}$$

and

$$\frac{{\text{dD}}_{\Delta }}{\text{dt}}+D_{\Delta }=d^{T}∙W_{d2km}^{+}\;D_{\Delta }\left(t\right)=-d^{T}(t)∙W_{d2km}^{+}$$

Which results in

$$\delta \left(t\right)=D_{\Delta }\left(t\right)-d_{\nabla }(t)=d^{T}\left(t\right)∙W_{d2km}^{+}+d^{T}\left(t\right)∙W_{d2km}^{-}=d^{T}(T)∙W_{d2km}$$

We have now corrected the differential equations and selected parameters in our methods as well – Equations (35) and (36).

Finally, the Methods section has a sizable number of matrices that have seemingly arbitrary entries.

The entries are not arbitrary as the non-zero entries and the sign of the entries are determined by connectivity considerations but it is true that the magnitudes of the non-zero values are somewhat arbitrary, having been chosen through hand tuning in order to (at least visually) match the recorded responses. After another reviewer #1 suggestion (point 5), we now provide Figure 14 —figure supplement 1, 2 and 3, which show how the reconstructed responses are affected by modifying these parameters.
